# A guide to laser microdissection‐based proteomics

**DOI:** 10.1111/febs.70653

**Published:** 2026-07-10

**Authors:** Cheng Shen, Kael Schoffer, Charlotte Williams, Maria C. Tanzer

**Affiliations:** ^1^ Walter and Eliza Hall Institute of Medical Research Parkville Australia; ^2^ University of Melbourne Parkville Australia

**Keywords:** Deep Visual Proteomics, FFPE tissue, laser microdissection, mass spectrometry, single‐cell proteomics, spatial proteomics

## Abstract

Laser microdissection‐based proteomics enables spatially resolved profiling of the proteome within intact tissue. Recent advances in histology, image analysis, laser microdissection and mass spectrometry have markedly increased the capability of this technology and driven the development of a range of powerful workflows. These now extend from regional tissue proteomics to emerging single‐cell applications, with subcellular proteome mapping on the horizon. In this review, we examine the current state of laser microdissection (LMD)‐based proteomics, outline what these approaches can offer, describe the standard workflow, and discuss which applications and instrumentation are best suited to address specific biological questions. We also highlight the strengths and limitations of current workflows and direct readers to key publications that describe and exemplify their successful implementation.

## Graphical abstract

This review takes researchers through the full laser microdissection proteomics workflow—from tissue preparation and image analysis to sample processing and mass spectrometry—offering practical solutions to common challenges at each step. Real‐world examples from across the field illustrate how the technology is being applied to disease tissue, making this an accessible and hands‐on guide for newcomers and experienced users alike.

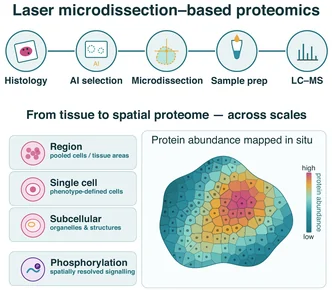

**Abbreviations**
ACN: acetonitrile
BIAS: Biology Image Analysis Software
DDM: n‐dodecyl‐β‐D‐maltoside
dia‐PASEF: data‐independent acquisition–parallel accumulation–serial fragmentation
DVP: Deep Visual Proteomics
FAIMS: field asymmetric ion mobility spectrometry
FFPE: formalin‐fixed and paraffin‐embedded
HIER: Heat‐Induced Epitope Retrieval
LC–MS: liquid chromatography–mass spectrometry
LMD: laser microdissection
MALDI‐MSI: matrix‐assisted laser desorption/ionisation mass spectrometry imaging
nanoPOTS: Nanodroplet Processing in One pot for Trace Samples
PEN: polyethylene naphthalate
PTM: post‐translational modification
SCP: single‐cell proteomics
TCEP: tris(2‐carboxyethyl)phosphine
timsTOF: trapped ion mobility spectrometry time‐of‐flight

## Introduction

Investigating spatially resolved protein regulation in tissues is essential for understanding cellular behaviour in its physiological context. While numerous microscopy‐based RNA technologies have been developed, measuring proteins *in situ* remains considerably more challenging [1, 2]. Antibody‐based imaging approaches, including instruments such as the MIBIscope platform, currently enable the simultaneous detection of up to ~40 proteins [3, 4]. MALDI‐MSI (matrix‐assisted laser desorption/ionisation mass spectrometry imaging) allows high spatial resolution detection of a broad range of molecules, including peptides; however, it remains limited in throughput and proteome depth [5]. An alternative strategy combines laser microdissection (LMD) of defined tissue regions, first described in 1996, with comprehensive protein analysis by mass spectrometry [6, 7]. Historically, however, this approach faced major limitations [8]. Technical constraints in LMD and, more importantly, the limited sensitivity of earlier generations of mass spectrometers restricted both spatial resolution and proteome depth. This landscape has changed dramatically in recent years, culminating in spatial proteomics being recognised as ‘Method of the Year 2024’ by Nature Methods [9]. Advances in LMD technology and, in particular, major improvements in mass spectrometry sensitivity now allow the analysis of very limited material while still achieving sufficient proteome depth to generate biologically interpretable data [10, 11, 12]. In parallel, automated cell and shape recognition approaches now enable the precise identification and isolation of specific cellular structures in a high‐throughput manner. These developments have recently been integrated into a unified workflow known as Deep Visual Proteomics (DVP), developed by the Mann laboratory [13]. This workflow enables proteomic analysis at single‐cell resolution and, more recently, even at subcellular scales or for the analysis of post‐translational modifications (PTMs) [14, 15, 16, 17, 18, 19]. While standard LMD proteomics workflows that use moderate amounts of material are now feasible in many experimental settings, analyses involving extremely small samples such as single cells, subcellular analyses or PTM‐focused workflows remain technically demanding and often require specialised instrumentation and expertise.

## When to use LMD‐based proteomics: Applications, strengths and limitations

Traditional approaches to unbiased proteomic profiling of specific cell populations *in situ* typically rely on tissue dissociation followed by flow cytometric cell sorting and downstream analysis. While powerful and high‐throughput, these methods disrupt tissue architecture and result in loss of spatial context. In addition, certain cell types and tissues are difficult to dissociate, and these approaches are often limited to fresh material, restricting their applicability to clinically relevant samples such as formalin‐fixed and paraffin‐embedded (FFPE) samples. LMD overcomes several of these limitations and is particularly well suited for analysing defined cell populations or regions within intact tissue. It enables selective isolation of cells based on morphology and spatial location and can be applied to archived material, including FFPE samples stored in biobanks [20, 21]. Crucially, compatibility with FFPE material extends the reach of LMD‐based proteomics to archived biobanks accumulated over decades, including internationally distributed collections. This is of particular benefit for rare diseases, where prospective recruitment of sufficient patient numbers is often impractical, and where access to historical specimens may be the only feasible route to achieving the sample sizes required for meaningful proteomic analysis [1, 22, 23].

Recent technological advances have expanded LMD‐based proteomics beyond the analysis of large regions or pooled cell populations (regular LMD‐based proteomics) (Fig. 1). Despite application‐specific differences and the introduction of new instrumentation, the overall workflow remains straightforward: Histology (a), AI‐driven image analysis and selection (b), LMD (c), sample preparation (d), and LC–MS‐based acquisition (e) (Fig. 1A–E). Recent improvements across these steps now enable single‐cell proteomics and tissue‐wide mapping, allowing spatial protein gradients to be detected and cellular organisation across tissues to be resolved based on hundreds to thousands of proteins per cell, rather than a limited number of markers [15]. Over time, several LMD platforms have been developed, including the Leica LMD7, Zeiss PALM MicroBeam, MMI CellCut and ArcturusXT, whose technical characteristics are compared in detail by Guo *et al*. [24] (Fig. 1C). In addition, a range of complementary technologies now support these workflows and increase throughput, including automated single‐cell dispensers such as the cellenONE and highly sensitive mass spectrometers such as the Bruker timsTOF SCP (trapped ion mobility spectrometry time‐of‐flight, single‐cell proteomics) and the Thermo Fisher Orbitrap Astral (asymmetric track lossless) mass analyser (Fig. 1D,E).

**Fig. 1:**
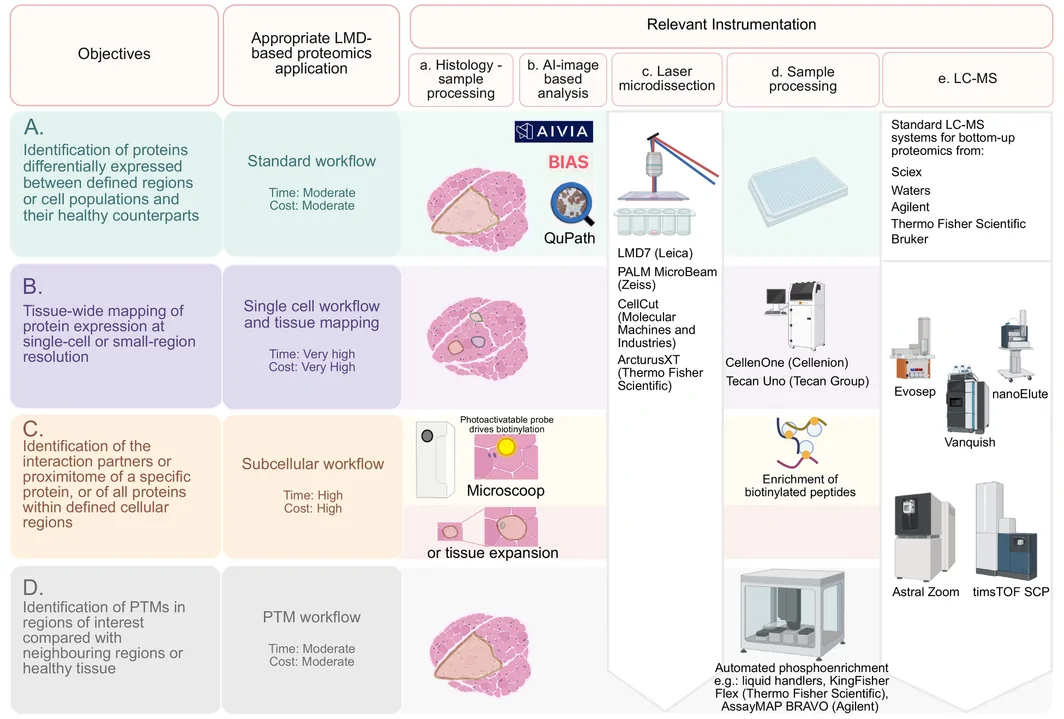
Overview of LMD‐based proteomics applications to address diverse biological questions, with examples of relevant instrumentation.

The next frontier is the analysis of subcellular structures, enabling the investigation of proteins within defined intracellular compartments that are otherwise difficult to isolate (Fig. 1C). However, LMD is limited by its cutting precision and inherently disrupts tissue architecture. To overcome these limitations, complementary approaches are emerging. Tissue expansion techniques can physically enlarge structures to make them accessible to LMD, while alternative strategies such as microscopy‐guided activation of photoactivatable probes by Syncell allow highly localised biotinylation of proteins within regions of interest [17, 25]. Additionally, combining LMD with phosphoproteomics is now feasible due to advances in highly sensitive phosphopeptide enrichment strategies (Fig. 1D) [16].

While these applications hold immense potential, several limitations and challenges remain within this technological platform and workflow (Fig. 2). Spatial information, for example, is not intrinsically encoded at high resolution but rather defined by the user during tissue selection. As such, LMD provides relatively coarse spatial resolution compared to imaging‐based or spatially barcoded approaches. Furthermore, while region selection can be supported or automated by AI‐driven image analysis such as AIVIA, BIAS (Biology Image Analysis Software) and QuPath, LMD remains time‐intensive and lower‐throughput than flow cytometry‐based approaches, particularly when small and numerous regions are isolated [13, 26, 27].

**Fig. 2:**
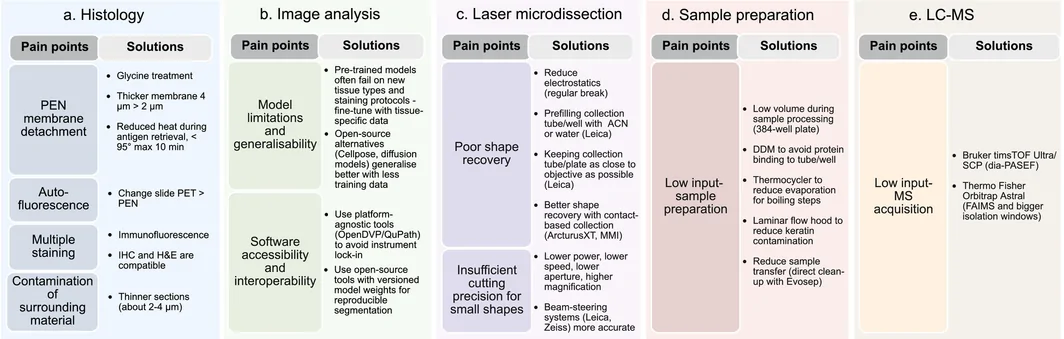
Summary of current pain points in the LMD‐based proteomics workflow and potential strategies to address these.

Certain tissue types (e.g. bone) or section properties (e.g. thickness) can further limit cutting efficiency. In addition, laser ablation can lead to sample loss, particularly when isolating very small regions. A further unresolved challenge is the loss of dissected material due to electrostatic effects during LMD (Fig. 2C). Repeated cutting increases electrostatic charge, which can prevent especially smaller fragments from correctly landing in the collection vessel. This contributes substantially to material loss and limits sensitivity. As a result, achieving meaningful proteome depth from single cells or subcellular regions remains challenging. Emerging approaches that combine LMD with local protein labelling strategies (e.g. proximity biotinylation) or tissue expansion techniques are promising but remain in early development [25]. These strategies will require careful optimisation of sensitivity and input requirements, as the performance of LMD‐based proteomics strongly depends on the amount of starting material. In practical terms, a conventional LMD experiment involving regional dissection of moderate‐sized areas can typically be completed within a standard working day, whereas single‐cell or tissue‐mapping workflows requiring hundreds to thousands of individual cuts may span multiple sessions. Instrument and reagent costs scale similarly. This is particularly relevant for single‐cell workflows, where multiplexing remains limited to relatively few channels, increasing acquisition time and cost [28]. Furthermore, standard LMD proteomics is accessible on widely available mass spectrometry platforms, while single‐cell and subcellular applications require specialised instrumentation that may not be available in all facilities (Fig. 1E). These practical considerations should inform workflow selection alongside biological requirements.

Overall, LMD proteomics is best applied when spatially defined, unbiased proteome analysis of specific cell populations or tissue regions is required—particularly for rare cell populations, small regions such as tumour niches, and histologically defined structures that would otherwise be inaccessible [14, 29, 30, 31, 32].

## LMD‐based proteomics workflow and its pain points

Detailed protocols covering all aspects of LMD sample preparation have already been described elsewhere [18, 33, 34]. Here, we outline the general workflow, recent advances, common pain points and practical ways to address them.

### Histology

LMD is most commonly applied to FFPE and fresh‐frozen material. Fresh‐frozen is generally considered better for preserving the native proteome, especially for phosphoproteomics [16]. However, disease samples are often more readily available as FFPE, and recent studies suggest that protein numbers can be broadly comparable between the two [21, 35].

As the first step in the LMD proteomics workflow, samples are mounted on membrane‐coated slides with the membrane composed of different materials such as PEN (polyethylene naphthalate), PPS (polyphenylene sulfide) and PET (polyethylene terephthalate). These membranes allow the UV laser to cut the selected tissue so it can be collected into the appropriate vessel. Samples can be prepared using histological methods such as H&E (haematoxylin and eosin), IHC (immunohistochemistry) or IF (immunofluorescence), enabling specific structures or proteins of interest to be stained. For optimal downstream staining, HIER (Heat‐Induced Epitope Retrieval) at 95°C is generally recommended; however, these conditions can damage membrane‐coated slides. Shortening the incubation time can reduce the risk of membrane detachment, but may also compromise staining quality (Fig. 2A). Therefore, staining and antigen retrieval conditions should be validated for each specific application. Work from the Coscia laboratory compared the advantages of HIER and PIER (pepsin‐induced antigen retrieval) methods for LMD slides [18]. While PIER has been reported to be gentler on the membrane, it tends to produce higher background staining. The addition of glycerol has also been reported to reduce membrane folding in PEN slides [36], while membrane thickness may further affect how well the membrane adheres to the glass slide.

Section thickness should be optimised during tissue preparation. While many FFPE tissues can be sectioned as thin as 2 μm, reducing contamination from adjacent regions, some materials, such as bone or fresh‐frozen tissue, are more difficult to cut thinly. Thinner sections improve spatial precision but reduce material recovery, whereas thicker sections provide more input material and may be easier to collect, particularly for small regions, but increase the risk of sampling unwanted tissue. The optimal thickness therefore depends on tissue type, region size, staining requirements and downstream proteomic sensitivity (Fig. 2A).

### Image analysis

High‐content imaging and artificial intelligence can be integrated with automated LMD to enable the systematic and higher‐throughput isolation of cells or tissue maps [32, 37]. DVP, for example, uses machine learning for automated cell segmentation and phenotypic classification, enabling precise excision of hundreds of phenotypically defined cells directly from their native tissue context—all with the instrument running autonomously [13, 38]. Positive cell detection and segmentation can be supported by a range of software tools, from commercial platforms such as BIAS [13] and AIVIA (Leica) to free, open‐source alternatives. OpenDVP extends this as a free, open‐source workflow in QuPath, using models such as Cellpose to identify positive cells and export .xml shape files for direct import into LMD software [27, 38, 39, 40] (Fig. 2B). More recently, ConvNeXt, a convolutional neural network developed for cellular morphology featurisation, enables automated phenotype‐guided cell selection and prediction of a cell's proteome directly from its visual appearance, paving the way for whole‐slide proteomics and more precise diagnostics [15]. Furthermore, emerging generative diffusion models have demonstrated greater robustness than conventional machine learning approaches while requiring less training data, making them a promising direction for future methodological development (Fig. 2B) [41].

### Laser microdissection

During LMD, laser power and aperture should be kept as low and narrow as possible to minimise damage to the sample and surrounding tissue, especially when isolating adjacent regions. A slower cutting speed can often compensate for these gentler settings, though thicker or tougher tissue may require higher power or a wider aperture for complete separation (Fig. 2C). UV‐based systems (Leica LMD7, Zeiss PALM MicroBeam, MMI CellCut) achieve faster cuts but generate more local heat than the infrared ArcturusXT, which causes less thermal damage to nucleic acids and is therefore preferred when RNA integrity is critical, particularly for FFPE material or single‐cell transcriptomics [24]. The Leica and Zeiss systems steer the laser beam while the slide remains stationary, offering greater positional accuracy and speed than the MMI CellCut and ArcturusXT, which move the stage instead.

Prolonged cutting can warm the slide and warp slide membranes, while electrostatic charge can cause detached sections to fold back onto the slide or miss the collection vessel; regular pauses therefore help maintain cutting accuracy. Susceptibility to static varies by collection mode: gravity‐dependent collection (Leica LMD7) and laser pressure catapulting (Zeiss PALM) are most affected by charge, humidity and temperature, whereas the contact‐based adhesive methods of the ArcturusXT (EVA thermoplastic film) and MMI CellCut (viscous cap) provide more reliable capture independent of these conditions. The collection vessel should be positioned as close as possible to the objective, and prefilling tubes with ACN (acetonitrile) further reduces electrostatic effects and improves collection efficiency, particularly for gravity‐ and catapulting‐based systems (Fig. 2C). For high‐throughput or multiplexed workflows, the Leica LMD7's compatibility with 96‐ and 384‐well plates offers a practical advantage over the single‐cap formats of the MMI and ArcturusXT systems.

### Sample preparation

Samples can be collected into a range of vessel formats, but lower volumes are thought to improve enzymatic efficiency for low‐input material, which is particularly important for single‐cell LMD (Fig. 2D). Common collection formats include 96‐well plates, 384‐well plates and chips. For very small inputs, such as single cells or subcellular regions, conventional protein quantification is not feasible. In these cases, the number of dissected cells or the total dissected area is used as a proxy for input amount. Under consistent tissue preparation conditions, protein yield scales approximately linearly with cell number, though the precise relationship depends on cell type, tissue composition, and section thickness [18]. Empirical calibration using defined cell numbers is therefore recommended when establishing a new workflow.

To address the challenge of limited sample input, new techniques have been developed for near‐lossless sample collection and processing, with minimising vessel transfers and use of low reaction volumes being key principles throughout the workflow. For example, the Nanodroplet Processing in One pot for Trace Samples (nanoPOTS), a well‐established platform coupled with LMD [38, 42], enables reaction volumes typically below 200 nL and has been shown to improve digestion kinetics. The LMD‐isolated tissue fragments are captured in low‐vapour‐pressure reagent droplets and then transferred by a robotic liquid‐handling platform into nanowells for downstream sample processing. A similar commercial platform, the cellenONE system coupled with proteoCHIP EVO 96 (Cellenion), is also widely used [39, 43, 44].

For low‐input and standard LMD, a widely used single‐pot lysis and digestion method, as described by Makhmut and colleagues among others, uses ACN and n‐dodecyl‐β‐D‐maltoside (DDM), with tris(2‐carboxyethyl)phosphine (TCEP) and chloroacetamide (CAA) included for reduction and alkylation, respectively [18, 42]. DDM is particularly important for low‐input samples, as it improves the recovery of hydrophobic peptides in proteomic workflows [45]. For FFPE samples, heating to 95°C is an essential step to reverse formalin crosslinks, which would otherwise modify peptides and affect their measured m/z during LC–MS. Because these workflows use very small volumes and include a high‐temperature step, careful control of evaporation is critical, and a thermocycler with a well‐sealed plate is generally recommended.

### LC–MS

While advances in sample preparation have been important, the development of increasingly sensitive liquid chromatography–mass spectrometry technologies has been the key driver enabling many biological applications. On the LC side, shorter gradients and improved chromatographic separation produce narrower, more intense peaks, which increases the signal of low‐abundance ions. Tip‐based LC systems such as the Evosep One further support this by minimising sample transfer between vessels, reducing sample loss at the point of injection [39, 46] (Fig. 2E).

Bruker released mass spectrometers well suited to single‐cell proteomics (timsTOF Ultra, timsTOF SCP), driven in part by their high‐capacity ion source, and paired these with optimised ion mobility windows in data‐independent acquisition–parallel accumulation–serial fragmentation (dia‐PASEF), providing a highly sensitive setup [12, 13, 47]. The Orbitrap Astral instruments are Thermo Fisher's counterpart platforms and combine field asymmetric ion mobility spectrometry (FAIMS) for removal of singly charged ions and improved signal‐to‐noise, a fast Astral analyser, and a highly sensitive detector. Notably, the use of wider isolation windows with lower input material allows greater accumulation of low‐abundance ions, improving sensitivity for limited samples. Additionally, the Zoom mode enables pre‐accumulation of ions in the bent trap prior to analysis, further enhancing detection of low‐abundance species [11, 48]. For samples with more material, standard mass spectrometry platforms are often sufficient (Figs 1E, 2E) [39, 47, 49].

## Applications

### Regular LMD‐based proteomics

LMD‐based proteomics has been successfully applied across a range of instrumental platforms. Although older setups may offer lower sensitivity, they can still generate biologically informative datasets when more material is available. Proteome depth is also influenced by factors beyond instrument choice, including tissue context and cell type, as shown by studies that used similar input amounts across different tissues and reported varying numbers of identified proteins [34]. Overall, conventional LMD‐based proteomics workflows routinely identify approximately 1500–8000 proteins from inputs ranging from 10 to thousands of cells across diverse instrumental configurations [50, 51, 52]. In the context of infection‐associated pathology, spatially resolved proteomic mapping has identified the left atrium as a site of heightened inflammatory vulnerability in COVID‐19‐affected cardiac tissue [53], and has elucidated how syphilitic infection disrupts epidermal homeostasis through degradation of the extracellular matrix niche that sustains epidermal stem cells [54]. In oncology, multi‐region profiling of breast cancer has demonstrated that proteomic heterogeneity increases with tumour grade through microenvironmental interactions rather than genomic changes [52]. When integrated with highly multiplexed imaging, LMD has revealed the distinct functional states of T cells compartmentalised by immunosuppressive macrophages in colorectal tumours. It has also characterised proteomic heterogeneity among cancer cells shaped by their spatial proximity to cytotoxic T cells [32]. In inflammatory disease, region‐specific dissection of kidney tissue has implicated the glomerulus as the primary locus of immune dysregulation in lupus nephritis [55] and has characterised the proteostatic impairment underlying defective muscle regeneration in inclusion body myositis [31]. Similarly, DVP of skin biopsies has identified type I and type II interferons as the key drivers of keratinocyte death and inflammation in toxic epidermal necrolysis, implicating the JAK/STAT signalling pathway as a central pathogenic axis and a potential therapeutic target [43]. Importantly, in toxicological research, spatial profiling of intestinal tissue has revealed that chronic alcohol exposure induces compartment‐specific pathological responses, specifically suppressing antimicrobial defences in crypts while triggering apoptosis and structural damage in villi [50]. Finally, validating LMD‐based proteomic findings is an important consideration, particularly given the limited material available and the technical variability inherent to low‐input workflows. Multiplexed imaging approaches—including multiplex immunofluorescence and highly multiplexed ion beam imaging—represent powerful complementary strategies for this purpose, enabling spatial confirmation of protein candidates at cellular resolution within intact tissue and directly linking proteomic discoveries to histological context. Targeted mass spectrometry can provide additional quantitative support. Such orthogonal validation is particularly valuable in rare disease settings, where small cohorts and limited material make replication in independent samples challenging [56].

### Single‐cell LMD‐based proteomics and tissue mapping

Following the success of bulk cell proteomics using LMD, increasing attention in this field has been directed toward the acquisition of minute regions of interest, especially at the single‐cell level. Biological samples are inherently heterogeneous at various levels, from outward phenotypes to intracellular genomics, transcriptomics and proteomics [13, 57, 58]. This heterogeneity necessitates the isolation of specific cell populations to avoid the “averaging effect” of bulk analyses, which can obscure distinct cellular states and mask critical functional shifts within particular cell groups. Conventional LMD generally depends on prior knowledge or the presence of clearly distinguishable tissue regions, such as those identified by H&E staining. Single‐cell or tissue‐mapping approaches can be performed without such prior assumptions. Systematic sampling across the tissue, coupled with the quantification of hundreds to thousands of proteins per segmented shape, enables unbiased mapping of protein distributions at relatively high spatial resolution and can reveal both fundamental organisational principles of tissue biology and disease‐associated alterations [15, 51]. However, important challenges remain. First, these analyses require precise cutting, extended cutting sessions and are impractical if dissection has to be performed manually. Second, the material obtained from a shape corresponding to a single cell contains only approximately 150 picograms of protein, making sample loss during ablation and processing the major barrier to achieving sufficient proteome coverage [13]. Technological advances across the LMD‐based proteomics workflow have recently made single‐cell LMD‐based proteomics feasible.

State‐of‐the‐art single‐cell proteomics approaches have demonstrated the capacity to identify upwards of 5300 proteins in individual A549 cells, averaging 3351 proteins in small cells and 4689 proteins in large ones [12, 28, 59]. However, workflows incorporating LMD typically yield considerably more modest proteomic coverage, with identifications ranging from 1000 to 4000 proteins [15, 18, 48, 60]. Low‐level multiplexing has been shown to improve the sensitivity of single‐cell proteomic analysis from LMD‐dissected samples [28].

In human liver tissue, single‐cell spatial proteomics has already revealed distinct proteomic signatures and limited intercellular interactions linked to intrinsic α1‐antitrypsin accumulation [15]. In parallel, refined cell selection strategies combined with continuous protein gradient mapping showed that around half of the proteins identified in individual hepatocytes across 18 participants exhibit zonated expression, while cross‐species analysis further highlighted features specific to human hepatic zonation [48]. A recent topographic LMD‐based proteomic study profiled an atypical teratoid‐rhabdoid tumour together with its adjacent tissue. This revealed spatially discrete molecular features at the tumour interface, including the boundary markers PYGL, ASPH and CD45 [51]. As tissue proteomic mapping through single‐cell LMD continues to advance, this approach holds considerable promise for deepening our understanding of tissue architecture and pathological processes.

### Subcellular LMD‐based proteomics

While most LMD‐based proteomics studies focus on protein expression in defined regions or single cells, there are important contexts in which subcellular localisation is critical. These include, for example, analysing host–pathogen interfaces, mapping synaptic contact sites, profiling important complexes such as the inflammasome, and comparing the proteome of defective versus intact organelles. In such settings, understanding protein distribution at the subcellular level provides key mechanistic insight.

Standard and single‐cell LMD‐based proteomics workflows are not well suited for these applications, particularly when targeting very small subcellular structures. Limited material leads to reduced proteome depth, often requiring the collection of many sites, while the cutting precision of LMD imposes inherent constraints. To address these challenges, complementary approaches have recently been developed to improve spatial resolution and sensitivity.

Firstly, tissue expansion—recently developed to improve the microscopic resolution of small structures—can also be applied to LMD. By physically expanding the tissue, structures of interest become more accessible, enabling more precise laser cutting and reducing contamination from surrounding regions. Importantly, this approach is compatible with FFPE tissue, making it particularly relevant for clinical samples [17].

An alternative strategy, which can be used independently of or in combination with LMD, has been developed by Syncell and is termed MicroScoop [25]. These approaches are tailored to the specific scientific question. The sample of interest, including FFPE tissue, is incubated with a photoactivatable probe that induces biotinylation of nearby lysine residues upon laser activation. This can isolate structures as small as 350 nm. By precisely directing the laser, proteins within defined regions can be selectively labelled. The entire tissue can then be processed, and biotinylated proteins enriched for downstream proteomic analysis. In this approach, the laser defines the spatial specificity of protein labelling. More targeted strategies aim to identify proteins that interact with, or are in close proximity to, a protein of interest. Syncell has developed an antibody‐conjugated photosensitizer, enabling local biotinylation of neighbouring proteins, increasing the precision to about 25 nm. These approaches avoid the requirement for genetic manipulation (e.g. TurboID constructs), investigate the proximitome of endogenously expressed proteins and allow direct application to tissue samples. A key challenge in these approaches is the relatively low abundance of biotinylated proteins or peptides compared to the high background of non‐labelled proteins during streptavidin‐based enrichment. Addressing this will require improved enrichment strategies and approaches to reduce background. One promising strategy is to combine spatially controlled biotinylation with LMD, thereby restricting analysis to regions or cells containing the protein or structure of interest and improving specificity.

### Post‐translational modifications in LMD‐based proteomics

To investigate spatially resolved signalling changes, recent efforts have aimed to combine LMD with enrichment of post‐translational modifications (PTMs). Although hundreds of different types of PTMs are known, LMD–PTM studies have so far focused on spatially resolved phosphorylation. Like other PTMs, phosphorylations are low in abundance. Even highly developed enrichment protocols often require substantial input material to achieve in‐depth phosphopeptide enrichment and confident phosphosite identification [61]. In addition to input amount, FFPE sample preparation and downstream processing are also less favourable for preserving phosphorylation and may therefore limit the performance of LMD‐based phosphoproteomics. Humphries *et al*. detected 2000–5000 phosphoprecursors from 30 μg of FFPE input material [35]. Although they observed a strong Pearson correlation between fresh‐frozen and FFPE samples in a second study, phosphoprecursor numbers were still significantly higher in fresh‐frozen material. Similarly, Zeneyedpour *et al*. reported fewer phosphosites in FFPE tissue than in fresh‐frozen samples [62]. Consistent with this, Oliinyk *et al*. showed that nanoPhos, the most advanced phosphoproteomic workflow reported so far, identified approximately 40 000 phosphorylation sites in fresh‐frozen tissue, but only 20 000 in matched FFPE samples, using 1 μg of input material [16]. A further consideration in low‐input phosphoproteomics is that Class I phosphosites, which are assigned with higher localisation confidence and are typically defined by a localisation probability of >75%, often represent only around half of all detected phosphosites.

Nevertheless, over the past few years, LMD‐based phosphoproteomics has been applied to a range of biological questions, particularly through workflows using the AssayMAP Bravo liquid handler for Fe^3+^‐NTA cartridge‐based phosphopeptide enrichment. Using 80 μg of starting material, Schweizer *et al*. (2023) identified up to 6000 phosphosites per sample and uncovered signalling changes in COVID‐19‐infected lung tissue [19]. Nordmann *et al*. reported around 16 000 phosphosites and characterised signalling alterations in toxic epidermal necrolysis skin [43], and Oliinyk *et al*. resolved brain region‐specific phosphoproteomes despite minimal input of 1000 shapes per sample [16]. These workflows remain exclusively bottom‐up; top‐down approaches capable of characterising intact proteoforms and combinatorial PTMs are an important future direction, but currently face fundamental barriers including insufficient sensitivity for low‐input material, poor chromatographic resolution of intact proteins, less efficient ionisation and fragmentation compared to peptides, and considerably less mature data analysis tools [63].

## Conclusion

LMD‐based proteomics has progressed rapidly and can now resolve spatial proteomes with remarkable depth, in some cases even at subcellular resolution. At the same time, important challenges remain. Sample loss during processing continues to limit sensitivity and reproducibility, particularly at very low input levels. The analysis of post‐translational modifications in such settings is still technically demanding, some cellular structures remain difficult to capture by LMD alone, and throughput is lower than for dissociation‐based approaches. Further advances in workflow optimisation, automation and scalability will therefore be essential for broader adoption.

Looking ahead, the full potential of LMD‐based proteomics will depend not only on continued technical refinement, but also on its integration with complementary technologies. Combining spatial proteomics with transcriptomics, metabolomics, lipidomics, PTM analysis or proximity‐labelling approaches will provide richer biological context and help address current limitations in sensitivity and interpretation [52, 64].

It is also worth noting that a number of the sources cited here are preprints. While this is not always standard practice, we chose to do so deliberately because these studies reflect important and emerging advances in the field. Together, they reflect the energy, creativity and rapid progress of this area of research. We believe this field has a particularly exciting future, with continued advances likely to deliver not only new technological capabilities, but also deeper biological insight and, ultimately, meaningful impact on our understanding of health and disease.

## Conflict of interest

The authors declare no conflict of interest.

## Author contributions

All authors contributed to the preparation of this manuscript.
